# A Novel Bispecific Antibody for EpCAM-Directed Inhibition of the CD73/Adenosine Immune Checkpoint in Ovarian Cancer

**DOI:** 10.3390/cancers15143651

**Published:** 2023-07-17

**Authors:** Emily Maria Ploeg, Isabel Britsch, Anne Paulien van Wijngaarden, Xiurong Ke, Mark Alexander Johannes Martinus Hendriks, Douwe Freerk Samplonius, Wijnand Helfrich

**Affiliations:** Department of Surgery, Laboratory for Translational Surgical Oncology, University of Groningen, University Medical Center Groningen, 9713 GZ Groningen, The Netherlands; e.m.ploeg@umcg.nl (E.M.P.);

**Keywords:** CD73, EpCAM, ovarian cancer, bispecific antibody, adenosine

## Abstract

**Simple Summary:**

Blockade of the immunosuppressive CD73/ADO immune checkpoint has been suggested as a promising alternate immunotherapeutic approach for refractory ovarian cancer (OC). Despite promising preclinical results, midterm clinical trial reports indicate that the efficacy of the CD73-blocking antibody oleclumab is modest. The limited efficacy of oleclumab may be related to the fact that it indiscriminately binds to and blocks CD73 that is present in a massive surplus of normal cells. The aim of our study was to achieve a tumor-directed inhibition of the CD73 immune checkpoint on OC cells. To this end, we constructed a novel bispecific antibody, bsAb CD73xEpCAM, that blocks the CD73 immune checkpoint in an EpCAM-directed manner. Moreover, treatment of OC cells with bsAb CD73xEpCAM inhibited various pro-oncogenic features. Taken together, bsAb CD73xEpCAM may be useful as an alternate and more tumor-directed immunotherapeutic approach to overcome the CD73-mediated immunosuppression in OC patients.

**Abstract:**

PD-1/PD-L1-inhibiting antibodies have shown disappointing efficacy in patients with refractory ovarian cancer (OC). Apparently, OC cells exploit nonoverlapping immunosuppressive mechanisms to evade the immune system. In this respect, the CD73-adenosine inhibitory immune checkpoint is of particular interest, as it rapidly converts pro-inflammatory ATP released from cancer cells to immunosuppressive adenosine (ADO). Moreover, cancer-cell-produced ADO is known to form a highly immunosuppressive extra-tumoral ‘halo’ that chronically inhibits the anticancer activity of various immune effector cells. Thus far, conventional CD73-blocking antibodies such as oleclumab show limited clinical efficacy, probably due to the fact that it indiscriminately binds to and blocks CD73 on a massive surplus of normal cells. To address this issue, we constructed a novel bispecific antibody (bsAb) CD73xEpCAM that inhibits CD73 expressed on the OC cell surface in an EpCAM-directed manner. Importantly, bsAb CD73xEpCAM showed potent capacity to inhibit the CD73 enzyme activity in an EpCAM-directed manner and restore the cytotoxic activity of ADO-suppressed anticancer T cells. Additionally, treatment with bsAb CD73xEpCAM potently inhibited the proliferative capacity of OC cells and enhanced their sensitivity to cisplatin, doxorubicin, 5FU, and ionizing radiation. BsAb CD73xEpCAM may be useful in the development of tumor-directed immunotherapeutic approaches to overcome the CD73-mediated immunosuppression in patients with refractory OC.

## 1. Introduction

Typically, tumors in patients with ovarian cancer (OC) are often diagnosed at an advanced stage [1]. Currently, cytoreductive therapy with platinum-based chemotherapy is the mainstay of disease management, to which the majority of patients with OC initially respond. Unfortunately, 70% of these patients eventually develop refractory recurrences [2]. Therefore, novel therapeutic approaches in advanced OC are urgently needed. In this respect, an immunotherapeutic anticancer approach based on immune checkpoint inhibitors appears promising because OC tumors are frequently populated with tumor-infiltrating lymphocytes (TILs). Moreover, the degree of TIL infiltration in OC tumors independently correlates with patient survival [3]. Clinical trials have been evaluating the activity of PD-1/PD-L1-inhibiting antibodies in relapsed OC patients. However, cancer patients may be unresponsive towards these inhibitors, as resistance includes the activation of the Wnt/β-catenin pathway [4], EGFR mutations and ALK rearrangements [5], and local immunosuppressive factors within the tumor microenvironment [6]. Moreover, treatment with PD-1/PD-L1-inhibiting antibodies also appears to upregulate other immune checkpoints [7]. Indeed, recent trials have reported only modest activity for PD-1/PD-L1-inhibiting antibodies in relapsed OC, with disappointing response rates of only 4% to 15% [8,9,10].

Apparently, OC exploits nonoverlapping immunosuppressive mechanisms to achieve immune evasion. In this respect, the CD73–adenosine inhibitory immune checkpoint is of particular interest [11]. Many cancer types, including OC, appear to exploit the CD73 overexpression to affect the immunosuppressive capacity of the tumor environment (TME). CD73 is an GPI-anchored ecto-5′-nucleotidase that is involved in the rapid conversion of pro-inflammatory extracellular ATP (eATP), released from cancer cells due to metabolic or therapy-induced stress, to adenosine (ADO), one of the most potent immunosuppressive regulatory molecules. In this process, eATP is rapidly metabolized by ectoenzyme CD39 to adenosine monophosphate (AMP), which is a substrate for rate-limiting hydrolysis to ADO by CD73 [12]. The produced ADO molecules form an immunosuppressive ‘halo’ in and beyond the TME that potently inhibits the anticancer activity of immune effector cells. Notably, high-level CD73 expression in the TME of OC cells was reported to abrogate the favorable prognosis associated with the presence of TILs [13].

Inhibition of the enzyme activity of CD73 is currently being explored as an alternative or complementary form of cancer immunotherapy [14]. Various CD73 antagonistic antibodies, the most notable being oleclumab (MEDI9447) [15], are currently undergoing clinical evaluation. Unfortunately, midterm reports on the treatment of relapsed OC patients with oleclumab alone or in combination with antagonistic PD-L1 antibody durvalumab indicate limited efficacy [16]. This lack of efficacy may be related to the fact that CD73 is broadly expressed on a wide variety of normal cells and tissues, potentially forming an antigen sink that precludes sufficient accretion of oleclumab at tumor lesions [17]. Moreover, generalized inhibition of the CD73 enzyme activity by oleclumab may potentially lead to autoimmune-related adverse events analogous to those observed for other clinically used immune checkpoint inhibitors.

Therefore, novel approaches are urgently needed to inhibit the OC-cell-expressed CD73 in a more tumor-directed manner. In this regard, bispecific antibodies (bsAbs) may improve the efficacy of cancer immunotherapy, as they can be engineered to more selectively inhibit immune checkpoints on OC cells [18]. Previously, we have reported superior antagonistic activity of bsAbs towards cancer cells that co-overexpress a tumor-associated antigen of interest and immune checkpoints PD-L1 [19,20] or CD47 [21,22], which was most likely attributable to the enhanced avidity associated with the tetravalent bsAb-format. To achieve an OC-directed blockade of the CD73/ADO immune checkpoint, we engineered a novel tetravalent bsAb, designated bsAb CD73xEpCAM, that selectively binds to the pan-carcinoma-associated cell surface antigen ‘Epithelial Cell Adhesion Molecule’ (EpCAM) and concurrently blocks CD73. We selected EpCAM for this purpose, as it is overexpressed in 55% to 75% of OC patients. Moreover, the EpCAM overexpression in these patients correlated with enhanced cancer cell proliferation, oncogenic signaling, chemoradiotherapy resistance [23,24], and decreased overall survival [24,25,26].

Here, we demonstrate that bsAb CD73xEpCAM has potent capacity to inhibit the CD73/adenosine immune checkpoint exposed on ovarian cancer cells in an EpCAM-directed manner. In particular, bsAb CD73xEpCAM restored the anticancer activity of T cells when incapacitated by OC-cell-produced ADO. Additionally, in vitro treatment of OC cells with bsAb CD73xEpCAM potently inhibited their proliferative capacity and sensitized them to the cytotoxic activity of cisplatin, doxorubicin, 5FU, and ionizing radiation.

## 2. Materials and Methods

### 2.1. Antibodies and Reagents

Antibodies: goat-anti-human-IgG APC (SouthernBiotech, Birmingham, AL, USA), mAbMM07 FITC (Sino Biological, Beijing, China), and EpCAM APC (Abcam).

Reagents/proteins/kits: fluorescent caspase 3/8-488 probe (Biotium), crystal violet staining (Abcam, Cambridge, UK), APCP (Sigma-Aldrich, Saint Louis, MO, USA), recombinant soluble human CD73 (sCD73) (Abcam, Cambridge, UK), recombinant soluble human EpCAM (sEpCAM) (Abcam, Cambridge, UK), CFSE CellTrace Far Red cell proliferation kit (Thermofisher, Waltham, MA, USA), colorimetric malachite green-based Pi assay kit (ab65622, Abcam, Cambridge, UK), T cell activation/expansion beads (Miltenyi Biotec, Bergisch Gladbach, Germany), and IFNγ ELISA kit (eBioscience, San Diego, CA, USA)

### 2.2. Cell Lines, Primary Patient-Derived OC Cells, and Transfectants

Cell lines OvCAR3, ES2, SKOV3, L37, SK-N-SH, A172, U87GM, and CHO-K1 cells were obtained from the American Type Culture Collection (ATCC, Manassas, VA, USA). L37.EpCAM is a rat (Wag/Rij) squamous-cell lung carcinoma cell line stably transfected with human EpCAM [27]. Cancer cells were cultured in DMEM or RPMI-1640 (Lonza, Bazel, Switzerland), supplemented with 10% fetal calf serum (FCS, Thermofisher, Waltham, MA, USA) at 37 °C in a humidified 5% CO2 atmosphere (unless specified otherwise). CHO-K1 cells were cultured in GMEM (First Link, Denver, CO, USA), supplemented with 5% dialyzed FBS (Sigma-Aldrich, Saint Louis, MO, USA). Primary patient-derived OC cells were collected during surgical resection. The use of remaining anonymous material is regulated under the code for good clinical practice in the Netherlands. Therefore, informed consent was waived in accordance with Dutch regulations.

CHO.CD73 cells stably expressing human CD73 were generated by lipofection (Fugene-HD, Promega, Madison, WI, USA) using a eukaryotic plasmid containing cDNA encoding human CD73 (Hygromycin B selection, Origene, Rockville, MD, USA). Likewise, CHO.EpCAM and L3745R.EpCAM cells were generated by lipofection using plasmids containing cDNA encoding human EpCAM (G418 selection, Sino Biological, Beijing, China).

OvCAR3 CD73-Knockout (KO) cells were generated using CRISPR-Cas9 gene editing technology by transfection of pSpCas9 BB-2A-GFP (PX458) plasmid (Addgene plasmid #48138) containing the CD73-targeting sgRNA 5′-GCAGCACGTTGGGTTCGGCG-3′ [28]. Likewise, OvCAR3 EpCAM-KO cells were generated by transfection of pSpCas9 BB-2A-GFP (PX458) plasmid containing the EpCAM-targeting sgRNA 5′-TAATGTTATCACTATTGATC-3′ [29]. Subsequently, OvCAR3-KO cells were enriched by negative MACS selection using MiniMACS columns (Miltenyi Biotech, 130-042-201, Bergisch Gladbach, Germany) and MACS microbeads (Miltenyi Biotech, 130-090-855, Bergisch Gladbach, Germany) according to standard protocol. KO of CD73 or EpCAM expression was evaluated by flowcytometry.

### 2.3. Construction of bsAb CD73xEpCAM-IgG2silent

DNA fragments encoding scFvCD73 and scFvEpCAM were generated by commercial gene synthesis service (Genscript, Piscataway, NJ, USA) based on published VH and VL sequence data. For construction and production of bsAb CD73xEpCAM-IgG2silent, we used eukaryotic expression plasmid pbsAb, which contains 3 consecutive multiple cloning sites (MCS). MCS#1 and MCS#2 are interspersed by a 22 amino acid flexible linker [30]. MCS#1 and MCS#2 were used for directional and in-frame insertion of DNA fragments encoding scFvCD73, scFvEpCAM, and MCS#3 for insertion of DNA fragments encoding human Fc IgG2s [19]. Analogously, pbsAb-CD73xMock-IgG2s encoding bsAb CD73xMock-IgG2s was constructed by replacing scFvEpCAM in pbsAb-CD73xEpCAM-IgG2s by scFvMCSP directed against CSPG4 [20,22]. Likewise, pbsAb-MockxEpCAM-IgG2s encoding bsAb MockxEpCAM-IgG2s was constructed by replacing scFvCD73 in pbsAb-CD73xEpCAM-IgG2s by scFv4-4-20 directed against fluorescein [21,22,31].

### 2.4. Production of Recombinant bsAbs

The Expi293 expression system (ThermoFisher) was used to produce bsAbs CD73xEpCAM-IgG2s, CD73xMock-IgG2s, and MockxEpCAM-IgG2s. Briefly, Expi293 cells were transfected with plasmid encoding the bsAb of choice and cultured on a shaker platform (125 rpm) at 37 °C, 8% CO2 for 7 d. Next, conditioned culture supernatant was harvested and cleared by centrifugation (4000× *g* for 30 min), after which bsAbs were purified using an HiTrap Mab-select column connected to an ÄKTA Start chromatography system (GE Healthcare Life Sciences, Chicago, IL, USA).

### 2.5. Assessment Dual Binding Activity of bsAb CD73xEpCAM

CHO, CHO.CD73, or CHO.EpCAM cells were incubated with increasing concentrations (0.01–10 µg/mL) of bsAbs CD73xEpCAM (or appropriate controls) at 4 °C for 45 min. Subsequently, cells were washed with PBS, after which cells were reincubated with an APC-labeled secondary goat-anti-human-Ig-antibody at 4 °C for 45 min. Binding data were acquired using Guava EasyCyte 6/2L flow cytometer (Merck Millipore, Burlington, MA, USA) and analyzed using GuavaSoft 3.2 software.

The binding of bsAb CD73xEpCAM (1 μg/mL) to OvCAR3 cells was accessed in the competing presence of sCD73, sEpCAM, or a combination thereof (each 10 μg) at 4 °C for 20 min. Binding of bsAb CD73xEpCAM to cancer cells was evaluated by flow cytometry using an APC-labeled goat-anti-human-Ig-antibody essentially as described above.

Binding of bsAb CD73xEpCAM (0.01–10 µg/mL) to L37 and L37.EpCAM rat cancer cells [19] was evaluated by flow cytometry using an APC-labeled goat-anti-human-Ig-antibody essentially as described above. Similar, binding of bsAb CD73xEpCAM (1 µg/mL) (or appropriate controls) to parental OvCAR3 and CD73-KO and EpCAM-KO variants thereof was evaluated by flow cytometry using an APC-labeled goat-anti-human-Ig-antibody essentially as described above.

Binding of bsAb CD73xEpCAM to OvCAR3 cells was assessed over time. In short, OvCAR3 cells were incubated for 30 s with bsAb CD73xEpCAM (1 μg/mL) (or appropriate controls) and washed with cold PBS. Next, cells were cultured at 37 °C and, at indicated time points, washed again with cold PBS. Binding of bsAb CD73xEpCAM to OvCAR3 cells was evaluated by flow cytometry using an APC-labeled anti-human-Ig-antibody essentially as described above.

### 2.6. Assessment of Internalization of bsAb CD73xEpCAM/Antigen Complexes

The capacity of bsAb CD73xEpCAM to internalize CD73 exposed on the cancer cell surface upon concurrent binding to EpCAM was assessed using OvCAR3 cells by flow cytometry. In short, cancer cells were incubated with bsAb CD73xEpCAM (1 µg/mL) (or controls) at 37 °C for 5 h, after which residual the presence of CD73 on the cancer cell surface was assessed using anti-CD73 mAbMM07. Of note, mAbMM07 binds to a nonoverlapping epitope on CD73 and as such does not interfere with the CD73-binding capacity of bsAb CD73xEpCAM.

### 2.7. Assessment Capacity of bsAb CD73xEpCAM to Inhibit the Enzyme Activity of CD73

Inorganic phosphate (Pi) formed during CD73-mediated hydrolysis of AMP to ADO was measured using a colorimetric malachite green-based Pi assay kit. In short, OvCAR3 cells were treated with bsAb CD73xEpCAM (1 µg/mL) (or appropriate controls) at 37 °C. At indicated time points, cells were washed with cold PBS and incubated at 37 °C for 24 h. Subsequently, cells were washed (20 mM HEPES, 120 mM NaCl, 5 mM KCl, 2 mM MgCl2, 10 mM Glucose, pH 7.4) to remove residual Pi-containing medium and were then incubated with AMP (100 µM, Sigma-Aldrich, Saint Louis, MO, USA) at 37 °C for 40 min. The supernatant was mixed with phosphate reagent, and color development was evaluated by measuring the absorbance at 650 nm using a microplate reader (VERSA max, Molecular Devices, San Jose, CA, USA) and corrected by subtracting background levels.

The % of enzyme inhibition was calculated according to the following formula:

$$\%\mathrm{of}\;\mathrm{CD}73\;\mathrm{enzyme}\;\mathrm{inhibition}=100-((\frac{X}{\mathrm{OD}650\max})*100)$$

where X = is the OD value measured in a given experiment minus the background (OD650_exp_ − OD650_background_); OD650_max_ is the amount of Pi present in the conditioned supernatant in the absence of bsAb.

The EpCAM-directed capacity of bsAb CD73xEpCAM to inhibit enzyme activity of CD73 exposed on the cancer cell surface was assessed using a panel of CD73^pos^/EpCAM^pos^ (OvCAR3, ES2, and SKOV3) and CD73^pos^/EpCAM^neg^ (SK-N-SH, A172, and U87GM) cell lines. In short, cancer cells were treated with bsAb CD73xEpCAM (1 µg/mL) (or appropriate controls) for 15 min, washed with cold PBS, and then incubated at 37 °C for 24 h. Similarly, OvCAR3 WT and EpCAM-KO cells were treated with increasing concentrations (0.01–10 µg/mL) bsAb CD73xEpCAM for 15 min, washed with cold PBS, and then incubated at 37 °C for 24 h. The CD73 enzyme activity was assessed essentially as described above.

The capacity of bsAb CD73xEpCAM to block enzyme activity of CD73 by OvCAR3 cells EpCAM-directed manner was also assessed in the competing presence of recombinant sEpCAM (10 µg) at 4 °C for 20 min. Subsequently, OvCAR3 cells were treated with bsAb CD73xEpCAM (1 µg/mL) for 15 min, washed with cold PBS and incubated at 37 °C for 24 h, and then assessed for inhibition of the enzyme activity of CD73 essentially as described above.

### 2.8. Assessment Capacity of bsAb CD73xEpCAM to Restore Proliferation Capacity of ADO-Suppressed T Cells

Peripheral Blood Mononuclear Cells (PBMCs) were labeled with CFSE-FarRed (Thermofisher, Waltham, MA, USA) according to manufacturer protocol. Next, CFSE-FarRed-labeled PBMCs were treated (or not) with bsAb CD73xEpCAM (1 µg/mL) (or appropriate controls) for 15 min, washed, and activated by addition of T cell activation/expansion beads (Miltenyi Biotec, Bergisch Gladbach, Germany) in a bead-to-cell ratio of 1-1 in medium supplemented (or not) with AMP (100 µM). Live cell imaging technology (IncuCyte, Sartoriusm Göttingen, Germany) was used to evaluate the size of T cell clusters by taking pictures at 4× magnification every 6 h for 7 d. The area (μm^2^/image) of activated T cell clusters was quantified using IncuCyte software 2019B.

### 2.9. Assessment Capacity of bsAb CD73xEpCAM to Restore Anticancer Activity of ADO-Suppressed T Cells

OvCAR3 cancer cells were treated (or not) with bsAb CD73xEpCAM (1 μg/mL) (or appropriate controls) for 15 min, washed and incubated in medium supplemented (or not) with AMP (100µM) for at 37 °C for 24 h. Next, freshly isolated PBMCs were added at different effector (E) to target (T) cell ratios to OvCAR3 cancer cells, and then co-cultured at 37 °C for 24 h. Subsequently, T cells present in the PBMC population were stimulated and redirected to kill OvCAR3 cancer cells using EpCAM-directed/CD3-agonistic bispecific antibody BIS-1 [32] at 37 °C for 24 h. Of note, at the concentration range used, binding of BIS-1 to OvCAR3 cancer cells is not impaired by bsAb CD73xEpCAM. Apoptotic cancer cell death was assessed by flow cytometry and shown as percentage of Annexin-V^pos^/PI^pos^ events.

### 2.10. Assessment Inhibitory Effect of bsAb CD73xEpCAM on Proliferation of Cancer Cells

OvCAR3 or ovarian carcinoma patient-derived cancer cells (8 × 10^3^/well) were treated with bsAb CD73xEpCAM (1 µg/mL) (or appropriate controls) for 15 min, washed, and then seeded onto an E-plate 16 (ACEA Biosciences, Santa Clara, CA, USA). Cell proliferation was monitored using the xCELLigence RTCA instrument (ACEA Biosciences, Santa Clara, CA, USA) at 37 °C for 60 h or 144 h.

OvCAR3 cancer cells (200/well) were treated with increasing concentrations (0.1–2 µg/mL) bsAb CD73xEpCAM (or appropriate controls) for 15 min, washed, and then cultured in a 6-well culture plate at 37 °C for 14 d. Next, cancer cell colonies were washed with PBS and stained with crystal violet. Cancer cell colony number and size were analyzed using ImageJ software (1.53t).

### 2.11. Assessment Sensitization of Cancer Cells by bsAb CD73xEpCAM for Chemotherapeutic Agents and Ionizing Radiation

OvCAR3 cancer cells (3 × 10^3^/well) were treated with bsAb CD73xEpCAM (1 μg/mL) (or appropriate controls) for 15 min, washed, seeded into a 96-well culture plate, and then incubated in the continuous presence (or absence) of 5FU (15 µg/mL), cisplatin (1 µg/mL), or doxorubicin (200 nM) at 37 °C for 4 d. Live cell imaging technology (IncuCyte) was used to assess proliferation of cancer cells by taking pictures at 4× magnification every 4 h for 5 d. Confluence (%) was measured using IncuCyte software 2019B.

OvCAR3 cells (200/well) were treated with (or without) bsAb CD73xEpCAM (1 µg/mL) (or appropriate controls) for 15 min, washed, and then incubated in the continuous presence (or absence) of 5FU (15 µg/mL), cisplatin (1 µg/mL), or doxorubicin (200 nM) in a 6-well culture at 37 °C for 14 d. Next, cancer cell colonies were washed with PBS and stained with crystal violet. Cancer cell colony number and size were analyzed using ImageJ software (1.53t).

OvCAR3 (200/well) were seeded in a 6-well culture plate, treated with (or without) bsAb CD73xEpCAM (1 µg/mL) (or appropriate controls) for 15 min, washed, irradiated with a 2 Gy dose of radiation, and then cultured at 37 °C for 14 d. Subsequently, cancer cell colonies were washed with PBS and stained with crystal violet. Colony number and size were analyzed using ImageJ software (1.53t). Irradiation (0.59 Gy/min) was performed using a 137Ce source (IBL 637 Cesium-137 γ-ray machine).

### 2.12. Statistical Analysis

Statistical analysis was performed by (multiple) *t*-test or two-way ANOVA followed by Tukey’s post hoc test, as indicated, using Prism software (8.0.1).

## 3. Results

### 3.1. BsAb CD73xEpCAM Has Dual Binding Specificity for OC-Exposed CD73 and EpCAM

**Figure 1:**
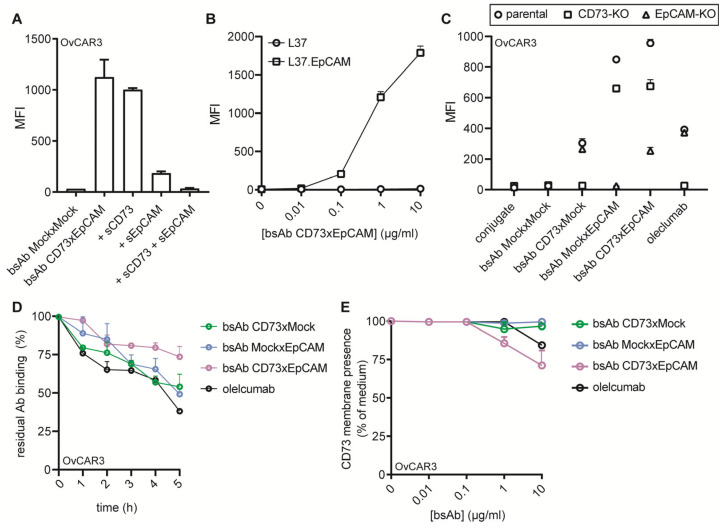
BsAb CD73xEpCAM has dual binding specificity for CD73 and EpCAM. (**A**) Competitive binding assay in which bsAb CD73xEpCAM (1 μg/mL) was pretreated with excess amounts of soluble CD73 (sCD73), soluble EpCAM (sEpCAM), or a combination thereof (10 μg) and then evaluated for binding to OvCAR3 cancer cells. (**B**) Dose-dependent binding of bsAb CD73xEpCAM to L37 and L37.EpCAM. (**C**) Binding of bsAb CD73xEpCAM (1 μg/mL) (or controls) to parental OvCAR3and corresponding CD73-KO and EpCAM-KO variants thereof. (**D**) Residual binding of bsAb CD73xEpCAM (1μg/mL) (or controls) to OvCAR3 cancer cells at indicated time points after 30 s of incubation and subsequent washing with PBS. (**E**) Residual CD73 membrane presence on OvCAR3 cells after treatment with bsAb CD73xEpCAM (0.01–10 µg/mL) (or controls) for 5 h. All experiments were analyzed by flow cytometry. All graphs represent mean ± SD.

The bsAb CD73xEpCAM was constructed in a so-called tetravalent bispecific taFv-Fc format (Figure S1). Its dual binding activity was confirmed using Chinese hamster ovary (CHO) cells that were transfected with either CD73 or EpCAM (Figure S2). The binding of bsAb CD73xEpCAM to OC cells was only slightly reduced in the presence of soluble CD73 (s.CD73), whereas soluble EpCAM (s.EpCAM) strongly inhibited the binding. The binding of bsAb CD73xEpCAM was fully abrogated in the combined competing presence of s.CD73 and s.EpCAM (Figure 1A), indicating that bsAb CD73xEpCAM selectively and simultaneously binds to CD73 and EpCAM. Moreover, bsAb CD73xEpCAM bound in a dose-dependent manner to rat cells transfected with human EpCAM (L37.EpCAM) and not to parental L37 cells (Figure 1B). Additionally, low binding of bsAb CD73xEpCAM was detected to CD73-KO and EpCAM-KO OC cells (Figure 1C). The binding analysis indicated that ~78% of bsAb CD73xEpCAM was still bound to OvCAR3 cells after 5 h, markedly outperforming the binding by oleclumab (~40% binding) (Figure 1D). Importantly, this difference in antibody binding is not due to the displacement of CD73 exposed on the cancer cell surface caused by the internalization of antibody/antigen complexes (Figure 1E).

### 3.2. BsAb CD73xEpCAM Treatment Potently Inhibits CD73 Enzyme Activity in an EpCAM-Directed Manner

**Figure 2:**
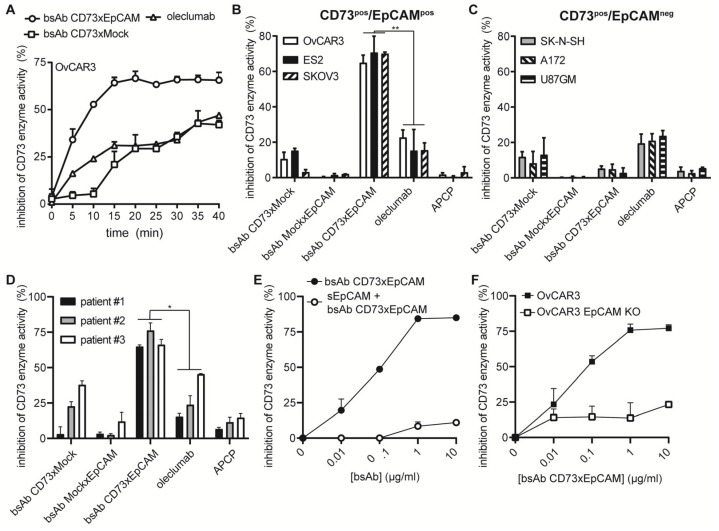
BsAb CD73xEpCAM potently inhibits the enzyme activity of CD73 in an EpCAM-directed manner. (**A**) Kinetics of inhibition of OvCAR3-exposed CD73 enzyme activity by bsAb CD73xEpCAM (1 μg/mL) (or controls). Inhibition of CD73 enzyme activity by treatment (15 min) with bsAb CD73xEpCAM (1 μg/mL) (or controls) of (**B**) CD73^pos^/EpCAM^pos^ cancer cell lines, (**C**) CD73^pos^/EpCAM^neg^ cancer cell lines, and (**D**) primary-OC-patient-derived carcinoma cells. (**E**) Competitive CD73 enzyme inhibition assay on OvCAR3 cells after treatment (15 min) with bsAb CD73xEpCAM in the presence of excess amounts of soluble EpCAM (sEpCAM). (**F**) Dose-dependent inhibition of CD73 enzyme activity by bsAb CD73xEpCAM (0.01–10 µg/mL) exposed on parental OvCAR3 cells versus OvCAR3 EpCAM-KO cells. CD73-mediated hydrolysis of AMP to ADO was evaluated using a colorimetric malachite green-based Pi assay. All graphs represent mean ± SD. Statistical analysis in (**B**,**D**) (group-mean) was performed using unpaired *t*-test (* *p* < 0.05, ** *p* < 0.01).

Treatment of OC cells with bsAb CD73xEpCAM rapidly inhibited the enzyme activity of CD73 exposed on the cancer cell surface, reaching its full inhibitory activity after 15 min (see Figure 2A, Figure S3 for experimental setup). In contrast, oleclumab reached its maximum CD73-inhibiting capacity only after 24 h (Figure S4). Accordingly, bsAb CD73xEpCAM significantly outperformed the CD73-inhibiting activity of both oleclumab and the small-molecule CD73-inhibitor APCP in three out of three CD73^pos^/EpCAM^pos^ OC cell lines (Figure 2B) but not in CD73^pos^/EpCAM^neg^ OC cell lines (Figure 2C, Figure S5). Importantly, bsAb CD73xEpCAM potently inhibited the CD73 enzyme activity in three out three CD73^pos^/EpCAM^pos^ primary-patient-derived OC cells (Figure 2D, Figure S6). Additionally, the capacity of bsAb CD73xEpCAM to inhibit the CD73-mediated hydrolysis of AMP was largely abrogated in the presence of excess amounts of s.EpCAM (Figure 2E) and when treating EpCAM-KO OC cells (Figure 2F).

### 3.3. BsAb CD73xEpCAM Treatment Overcomes ADO-Mediated Suppression of T Cell Proliferation

**Figure 3:**
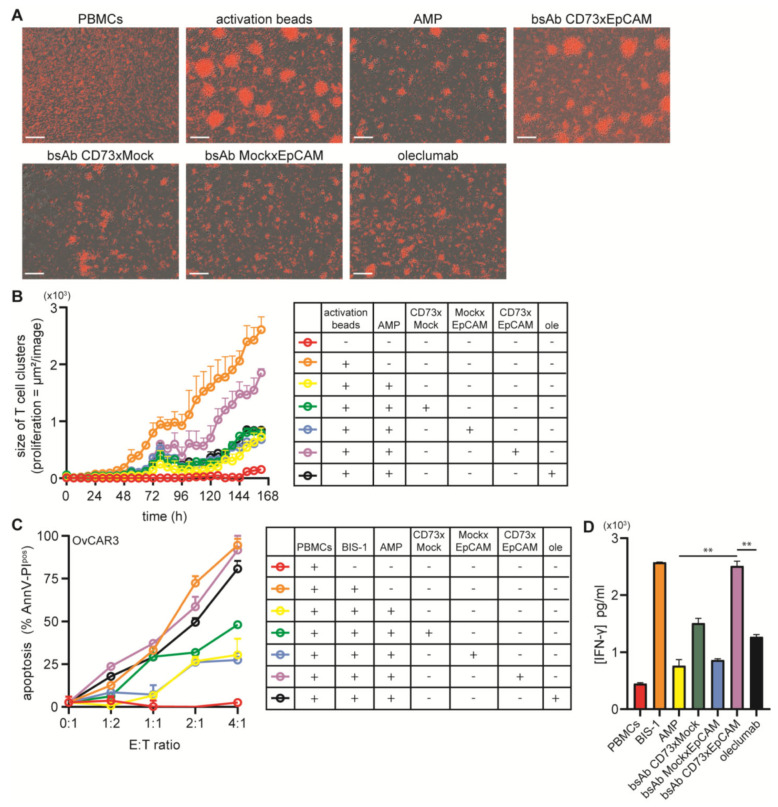
BsAb CD73xEpCAM restores the anticancer activities of ADO-suppressed T cells. (**A**) Representative images of activated CFSE-FarRed-labeled PBMCs treated (15 min) (or not) with bsAb CD73xEpCAM (1 µg/mL) (or controls), washed, and then cultured in medium supplemented with AMP at 37 °C for 7 d. Scale bar = 400 µm. (**B**) Cluster size (µm^2^/image) of activated proliferating T cell quantified using live cell imaging by taking pictures at 4× magnification at 37 °C every 6 h. (**C**) Percentage of AnnexinV-PI^pos^ (apoptotic) OvCAR3 cancer cells treated (15 min) with bsAb CD73xEpCAM (1 µg/mL) (or controls), washed, and then cultured in medium supplemented with AMP at 37 °C for 24 h. Subsequently, PBMCs were redirected to kill OvCAR3 cancer cells at increasing effector (E) to target (T) cell ratios. (**D**) IFN-γ levels excreted in culture supernatant (**C**) were measured by ELISA. All graphs represent mean ± SD. Ole = oleclumab in graphs B and C. Statistical analysis in D was performed using unpaired *t*-test (** *p* < 0.01).

When CFSE-FarRed-labeled T cells were subjected to AMP, which is enzymatically converted to ADO by CD73, and subsequently stimulated to proliferate using T cell activation beads, their proliferative capacity was significantly repressed, as can be appreciated from the decrease in the size of T cell clusters (see Figure S7 for experimental setup). Importantly, treatment of PBMCs with bsAb CD73xEpCAM fully overcomes the ADO-mediated inhibition of T cell proliferation, whereas oleclumab failed to do so (Figure 3A). These results corroborate the concurrent increase in clusters of activated T cells over time (Figure 3B).

### 3.4. BsAb CD73xEpCAM Treatment Restores Anticancer Activity of ADO-Suppressed Cytotoxic T Cells

When cytotoxic T cells were subjected to AMP and subsequently redirected to kill EpCAM-expressing OC cells using EpCAM-directed/CD3-antagonistic bispecific antibody BIS-1, induction of cancer cell death, evident from high caspase-3/8 activation levels in target cells, significantly dropped (see Figure S8 for experimental setup). Importantly, treatment of OC cells with bsAb CD73xEpCAM fully restored the cancer cell killing capacity of ADO-suppressed cytotoxic T cells (Figure 3C). These results corroborated ELISA data quantifying the restoration of the capacity of ADO-suppressed cytotoxic T cells to secrete IFN-γ (Figure 3D).

### 3.5. BsAb CD73xEpCAM Treatment Inhibits the Proliferative and Colony-Forming Capacity of OC Cells

**Figure 4:**
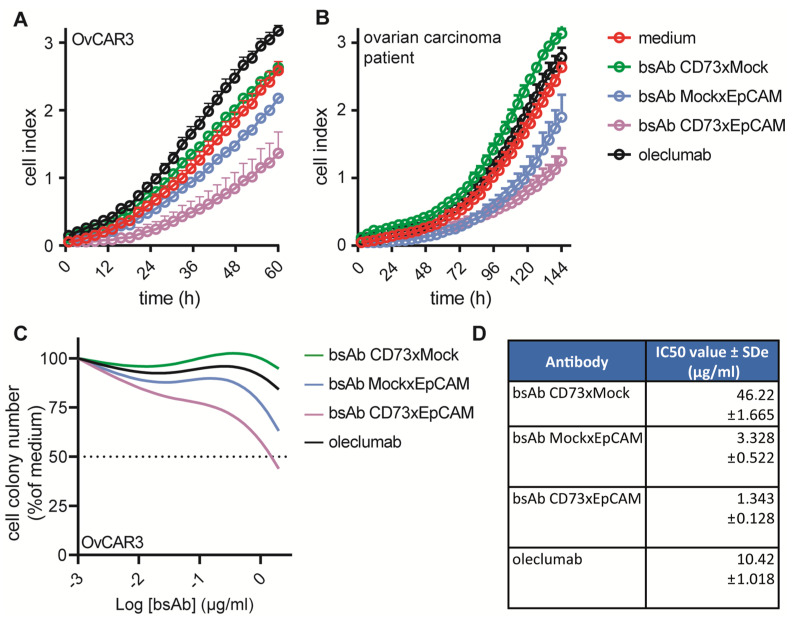
BsAb CD73xEpCAM inhibits the proliferative capacity of EpCAM-expressing cancer cells. Proliferation of (**A**) OvCAR3 and (**B**) primary-OC-patient-derived carcinoma cells after treatment with bsAb CD73xEpCAM (or controls) (1 μg/mL), using the RTCA xCELLigence instrument. The readout is indicated as cell-index, an arbitrary unit for attachment of adherent cells and cell proliferation measured at 37 °C every 15 min. (**C**) Inhibition of colony formation by OvCAR3 after treatment (15 min) with bsAb CD73xEpCAM (0.1–2 μg/mL) (or controls) and cultured at 37 °C for 14 d. (**D**) Table with IC50 values (μg/mL) calculated for graph (**C**). All graphs represent mean ± SD.

Both CD73 and EpCAM overexpression was reported to be implicated in the enhancement of cancer cell proliferation [33,34] and in resistance towards cytotoxic agents [23,24,35,36]. In this respect, it is encouraging that treatment of OC cells (Figure 4A) and primary-OC-patient-derived carcinoma cells (Figure 4B) with bsAb CD73xEpCAM significant decreased their proliferative and colony-forming capacity (Figure 4C and Figure S9). In particular, the corresponding colony-forming IC50 value calculated for bsAb CD73xEpCAM (1.343 μg/mL) was superior compared to that of oleclumab (10.42 μg/mL) (Figure 4D).

### 3.6. BsAb CD73xEpCAM Treatment Sensitizes OC Cells towards Chemotherapeutic Agents and Ionizing Radiation

**Figure 5:**
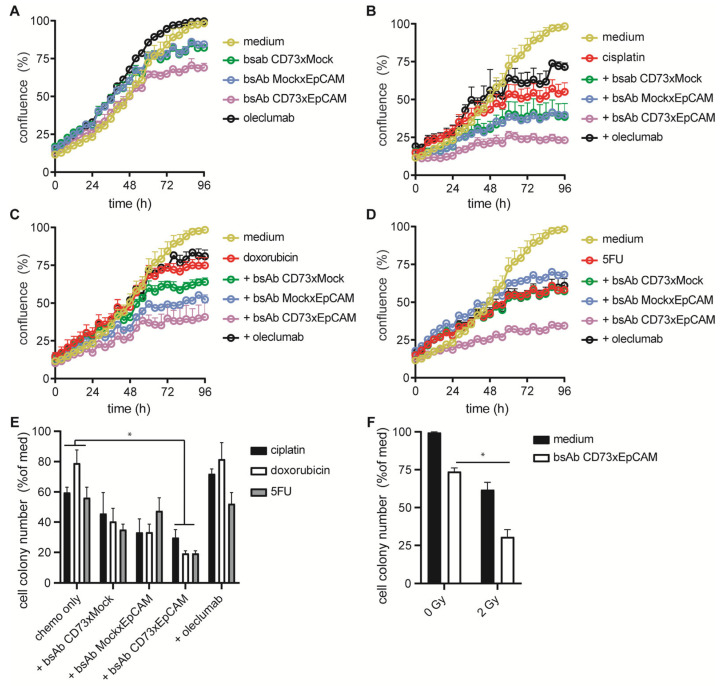
BsAb CD73xEpCAM sensitizes OvCAR3 cancer cells towards chemo- and radiotherapy. OvCAR3 cell confluence after pretreatment (15 min) with (**A**) bsAb CD73xEpCAM (1 μg/mL) (or controls) and subsequent treatment with (**B**) cisplatin (1 µg/mL), (**C**) doxorubicin (200 nM), or (**D**) 5FU (15 μg/mL). Cell confluence was evaluated using live cell imaging technology by taking pictures at 4× magnification every 4 h for 4 d. (**E**) Percentage of OvCAR3 cell colonies after pretreatment (15 min) with bsAb CD73xEpCAM (1 µg/mL) (or controls) and subsequent treatment with 5FU (15 µg/mL), cisplatin (1 µg/mL), or doxorubicin (200 nM) at 37 °C for 14 d. (**F**) Percentage of OvCAR3 cell colonies after pretreatment (15 min) with bsAb CD73xEpCAM (1 µg/mL), irradiated (or not) with 2 Gy and subsequently cultured at 37 °C for 14 d. All graphs represent mean ± SD. Statistical analysis in E (group-mean) and F was performed using unpaired *t*-test (* *p* < 0.05).

In vitro treatment of OC cells with bsAb CD73xEpCAM sensitized these cells towards the cytotoxic activity of cisplatin, doxorubicin, and 5FU. In particular, single treatment of OC cells with bsAb CD73xEpCAM decreased cell confluence by ~30% (Figure 5A), which decreased further when combined with cisplatin (~66%, Figure 5B), doxorubicin (~66%, Figure 5C), or 5FU (~53%, Figure 5D). Additionally, co-treatment of bsAb CD73xEGFR with cisplatin, doxorubicin, or 5FU significantly reduced the colony-forming capacity of OC cells (Figure 5E). Similarly, co-treatment with bsAb CD73xEGFR enhanced sensitivity of OC cells towards cytotoxicity induced by ionizing radiation up to ~45% (Figure 5F).

## 4. Discussion

Antibodies that inhibit the PD-1/PD-L1 immune checkpoint have shown limited clinical benefit in patients with advanced OC [8,9,10]. This reduced efficacy may be attributable (in part) to the highly immunosuppressive TME and low PD-L1 expression in OC tumors. Additionally, OC cells may exploit alternate and/or additional inhibitory immune checkpoints to achieve immune evasion and subsequent malignant progression. In this respect, the immunosuppressive cell-surface nucleotidase CD73 is of particular interest, as it is implicated in tumor progression and metastasis in OC. Moreover, CD73 inhibitors act on a variety of immune effector cells, while the effects of PD1/PD inhibitors are mostly limited to T cells. In line with this, CD73-targeting therapies may not be affected by all mechanisms that cause resistance to PD-1/PD-L1 inhibitors, and a larger subset of (OC) patients might benefit from this treatment modality.

High levels of CD73 have been shown to be negatively correlated with disease-free survival and overall survival rates in patients with high-grade serous (HGS) OC. Furthermore, high CD73 expression levels in OC tumors abrogated the favorable prognosis associated with increased levels of intraepithelial CD8^pos^ cytotoxic T cells. Moreover, in vitro studies have indicated that both elevated CD73 expression and enhanced extracellular ADO levels promote OC tumor growth and induce the expression of antiapoptotic BCL-2 family members in OC cells [13].

Several preclinical studies suggested that CD73 is a promising target for cancer treatment. Currently, several multicenter trials are ongoing to evaluate the clinical potential of the antagonistic CD73-antibody oleclumab [15] in patients with advanced solid malignancies, including OC. However, recent midterm reports indicate that as a single treatment modality the efficacy of oleclumab remains modest at best [24,27,37,38]. Clearly, novel strategies are urgently needed to enhance the clinical impact of CD73-inhibiting antibodies in OC patients.

Bispecific antibodies (bsAbs) are a promising class of immunotherapeutics with the potential to improve the clinical efficacy and safety of immune-checkpoint-inhibiting approaches. BsAbs can be engineered to selectively target, modulate, and interconnect biologic activities of otherwise separately acting surface receptors and ligands in a predesigned manner [39]. Moreover, tetravalent bsAbs are known to have significantly enhanced avidity towards cells that simultaneously express both targets antigens of interest, as they have up to four binding sites available for enhancement of functional interactions [40]. Importantly, an increasing number of bsAb-based immunotherapeutics are currently being evaluated in preclinical and clinical studies. To achieve the OC-directed inhibition of the CD73–ADO immune checkpoint, we produced bsAb CD73xEpCAM, which was constructed in a tetravalent bispecific taFv-Fc format [15]. We selected the clinically relevant pan-carcinoma target antigen EpCAM for this purpose, as it is selectively overexpressed in 55%-75% of OC patients.

In our study, the bsAb CD73xEpCAM outperformed oleclumab with respect to its binding capacity to CD73 exposed on EpCAM^pos^ OC cells. After 5 h, ~78% of the initial cell-bound fraction of bsAb CD73xEpCAM remained bound to OC cells in the presence of serum at 37 °C. In contrast, under identical conditions, only ~40% of oleclumab was still bound to the cancer cell surface. Previously, Hey et al. [15] demonstrated that oleclumab can displace CD73 from the cell surface by driving the internalization of antibody/antigen complexes. Therefore, we investigated whether or not the cell-surface displacement of CD73 induced by oleclumab would drastically affect the results of our binding analyses. This investigation indicated that after treatment with oleclumab for 5h the presence of CD73 on the cell surface was only moderately reduced by ~18%.

Treatment of OC cells with bsAb CD73xEpCAM resulted in potent inhibition of CD73 exposed on the cancer cell surface to convert extracellular AMP to ADO. The maximum inhibition of the OC-cell-exposed CD73 enzyme activity by bsAb CD73xEpCAM was reached after only 15 min, whereas oleclumab and bsAb CD73xMock required 24 h to do so. Moreover, bsAb CD73xEpCAM outperformed oleclumab in inhibiting the CD73 enzyme activity in three out of three primary-patient-derived OC cells. This remarkable activity of tetravalent bsAb CD73xEpCAM is most likely attributable to its enhanced avidity for binding to EpCAM exposed on the cancer cell surface and the concurrent blocking of the CD73 activity. Previously, we reported similar attributes for analogous tetravalent bsAbs designed to inhibit immune checkpoints PD-L1 [19,20], CD47 [21,22], and CD73 (bsAb CD73xEGFR, manuscript under revision, JITC, Ploeg et al.) in a tumor-directed manner.

It is well established that EpCAM overexpression is associated with increased cancer cell proliferation [41]. In this respect, it is noteworthy that in vitro treatment with bsAb CD73xEpCAM potently inhibited the proliferative capacity of both OvCAR3 cells and CD73^pos^/EpCAM^pos^ primary-patient-derived OC cells by ~49% and 56%, respectively. Moreover, bsAb CD73xEpCAM showed potent capacity (IC50 1.3 µg/mL) to inhibit the colony-forming capacity of OC cells, which was superior compared to that of oleclumab (IC50 10.4 µg/mL), bsAb CD73xMock (IC50 46.2 µg/mL), and bsAb MockxEpCAM (IC50 3.3 µg/mL).

Both CD73 and EpCAM overexpression was reported to be associated with enhanced resistance to chemotherapeutic agents and ionizing radiation [23,24,35,36]. Remarkably, platinum-based chemotherapeutic agents appear to enhance the CD73 expression levels in OC cells [19]. Additionally, platinum-based chemotherapeutic agents preferentially eliminate EpCAM-negative OC cells, suggesting that the remaining EpCAM-positive cells may contribute to tumor recurrence after chemotherapy [42]. In this respect, it is encouraging that in vitro treatment with bsAb CD73xEpCAM sensitized CD73^pos^/EpCAM^pos^ OC cells to the cytotoxic activity of cisplatin, doxorubicin, and 5FU by ~66%, ~66%, and ~53%, respectively. Similarly, in vitro treatment of OC cells with bsAb CD73xEpCAM enhanced their sensitivity towards radiation-induced cytotoxicity by ~25%. These results appear in line with the data that indicate that the siRNA-mediated silencing of EpCAM and CD73 enhances sensitivity to chemotherapeutic agents and ionizing radiation in breast, pancreatic, and prostate cancer cells [35,43,44,45].

## 5. Conclusions

In conclusion, bsAb CD73xEpCAM harbors multiple and possibly mutually reinforcing anticancer activities, including (1) enhanced binding avidity for CD73^pos^/EpCAM^pos^ OC cells; (2) potent and EpCAM-dependent inhibition of the CD73 enzyme activity of (primary-patient-derived) OC cells; (3) restoration of ADO-mediated suppression of T cell proliferation; (4) restoration of the anticancer activity of ADO-suppressed cytotoxic T cells; (5) inhibition of the proliferative capacity of (primary-patient-derived) OC cells; and (6) sensitization of OC cells to chemotherapeutic agents and ionizing radiation. Taken together, bsAb CD73xEpCAM may be useful to devise an alternate and more tumor-selective immunotherapeutic approach to overcome the CD73-mediated immunosuppression in patients with EpCAM-overexpressing refractory OC.

## Data Availability

All data relevant to this study are included in the article or uploaded as supplementary information.

## Supplementary Materials

The following supporting information can be downloaded at https://www.mdpi.com/article/10.3390/cancers15143651/s1, Figure S1: bsAb CD73xEpCAM; Figure S2; bsAb CD73xEGFR has dual binding specificity for CD73 and EGFR; Figure S3: Experimental procedure with CD73 enzyme assay; Figure S4: bsAb CD73xEpCAM rapidly inhibits the enzyme activity of CD73, outperforming oleclumab; Figure S5: CD73 and EpCAM expression levels on 8 cell lines; Figure S6: CD73 and EpCAM expression levels on patient-derived OC cells; Figure S7: Experimental procedure with T cell proliferation; Figure S8: Experimental procedure with cancer cell elimination by T cells; Figure S9: bsAb CD73xEpCAM inhibits the colony-forming activity of OvCAR3 cancer cells.
