# Supplementary material for: A Novel Bispecific Antibody for EpCAM-Directed Inhibition of the CD73/Adenosine Immune Checkpoint in Ovarian Cancer
Source: Cancers (Basel). 2023 Jul 17;15(14):3651. doi: 10.3390/cancers15143651 (PMC10378099; doi:10.3390/cancers15143651)
Supplement: Supplementary file 1 [file cancers-15-03651-s001.zip › cancers-2458315-supplementary.pdf]

SUPPLEMENTARY DOCUMENTATION

A novel bispecific antibody for EpCAM-directed inhibition of the CD73/adenosine immune checkpoint in ovarian cancer

Emily Maria Ploeg, Isabel Britsch, Anne Paulien van Wijngaarden, Xiurong Ke, Mark Alexander Johannes Martinus Hendriks, Douwe Freerk Samplonius and Wijnand Helfrich *

Department of Surgery, Laboratory for Translational Surgical Oncology, University of Groningen, University Medical Center Groningen, 9713 GZ Groningen, The Netherlands

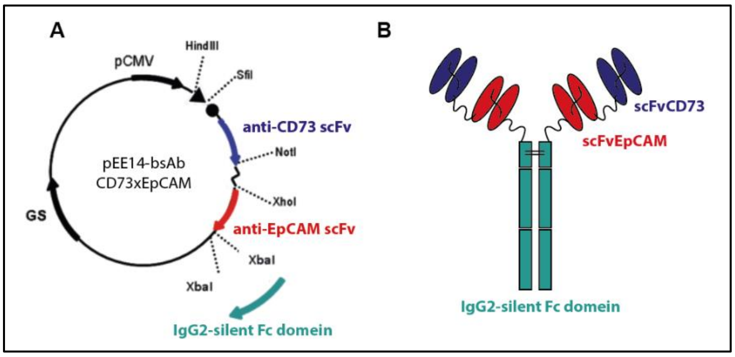

Supplementary Figure S1: bsAb CD73xEpCAM
(A) Topology of expression plasmid pbsAb encoding bsAb CD73xEpCAM-IgG2s and (B) schematic depiction of bsAb CD73xEpCAM-IgG2s protein.

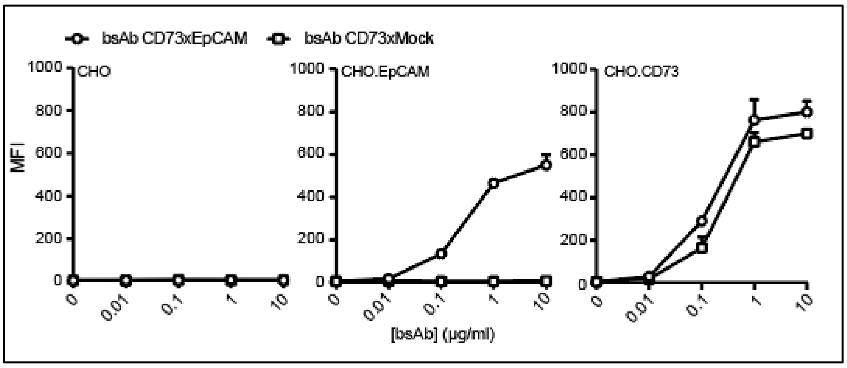

Supplementary Figure S2: bsAb CD73xEGFR has dual binding specificity for CD73 and EGFR
Dose-dependent binding of bsAb CD73xEGFR and bsAb CD73xMock to CHO, CHO.hEGFR, and CHO.hCD73, respectively. Experiments were analyzed by flow cytometry. All graphs represent mean ± SD.

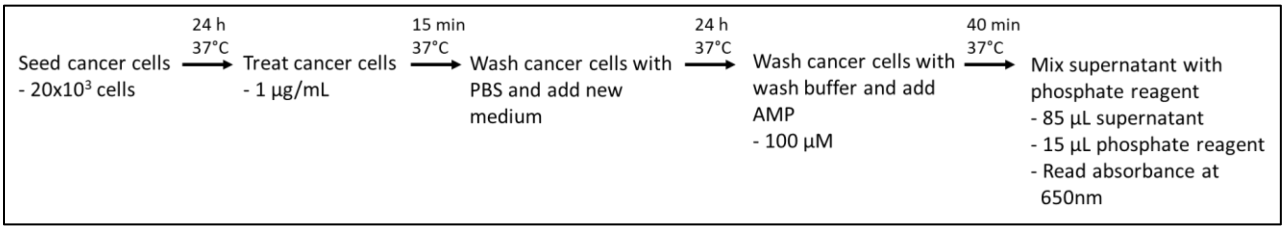

Supplementary Figure S3: Experimental procedure CD73 enzyme assay
Cancer cells were treated with bsAb CD73xEpCAM (1 μg/mL) (or appropriate controls) at 37°C for 15 min, washed with cold PBS, and incubated at 37°C for 24 h. Subsequently, cells were washed (20 mM HEPES, 120 mM NaCl, 5 mM KCl, 2 mM MgCl2, 10 mM Glucose, pH 7.4) to remove residual Pi-containing medium and were then incubated with AMP (100 μM) at 37°C for 40 min. The supernatant was mixed with phosphate reagent and color development was evaluated by measuring the absorbance at 650 nm using a microplate reader.

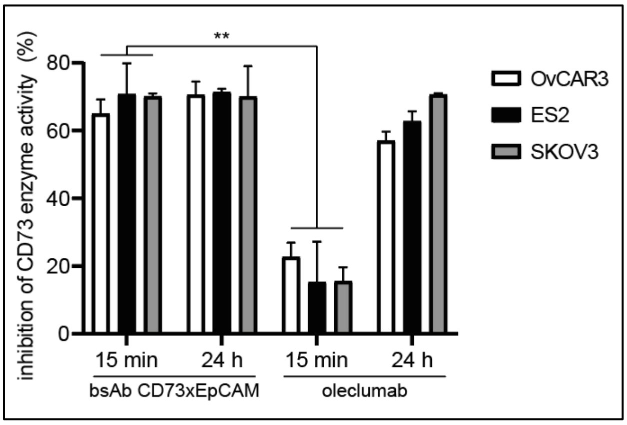

Supplementary Figure S4: bsAb CD73xEpCAM rapidly inhibits the enzyme activity of CD73, outperforming oleclumab
*Percentage inhibition of CD73 enzyme activity on a panel of CD73^{pos}/EpCAM^{pos} OC cell lines after treatment (15 min or 24 h) with bsAb CD73xEpCAM or oleclumab (both 1 μg/ml). Graph represent mean ± SD. Statistical analysis (group-mean) was performed using unpaired T-test (**p < .01).*

A

Cell line	MFI CD73	MFI EpCAM
OvCAR3	17.18	392.55
SKOV3	16.34	204.31
ES-2	19.84	16.42
SK-N-HS	64.33	1
U87MG	25.47	4.22
A172	25.91	0.57

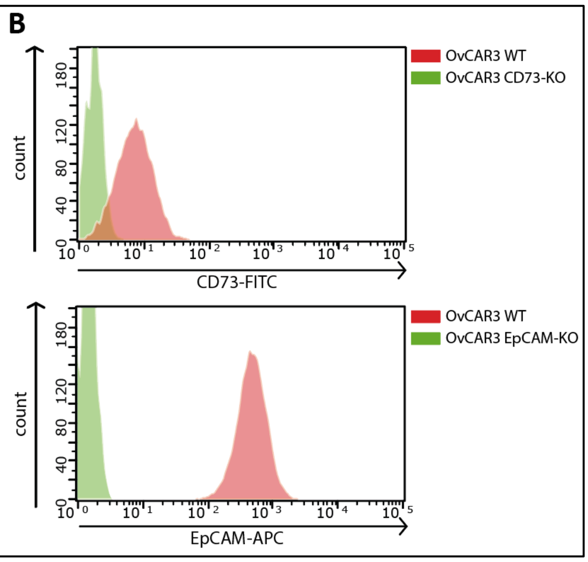

Supplementary Figure S5: CD73 and EpCAM expression levels on 8 cell lines
(A) Mean fluorescence intensity (MFI) of CD73 and ECAM expression levels on a panel of 6 cell lines used in this study. (B) Representative histogram of CD73 and EpCAM expression levels on OvCAR3 WT vs CD73- or EpCAM-KO OC cells. Expression levels were analyzed by flow cytometry.

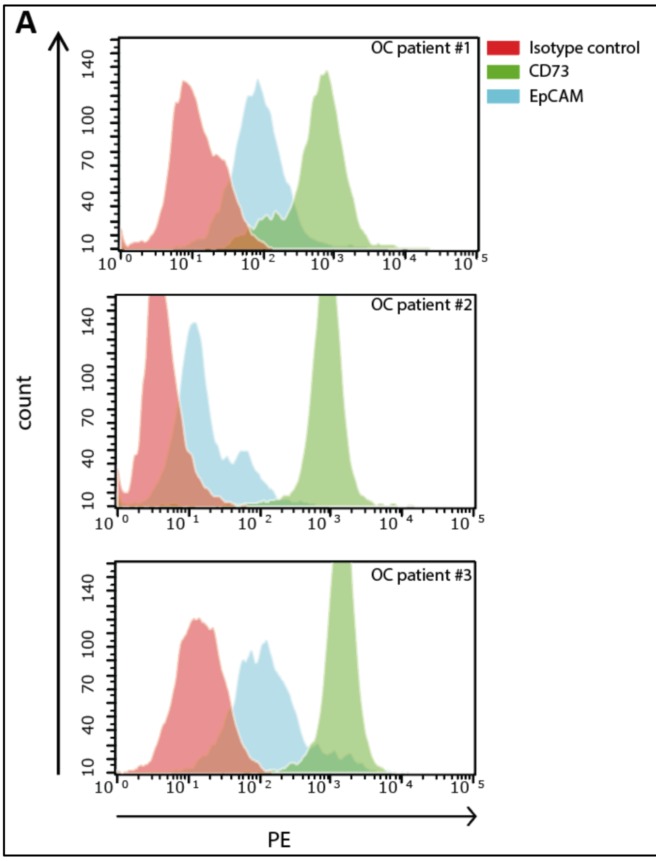

B

OC patient number	MFI CD73	MFI EpCAM
1	564.59	91.18
2	801.74	18.26
3	1361.82	16.57

Supplementary Figure S6: CD73 and EpCAM expression levels on patient-derived OC cells.
(A) Representative histograms of patient-derived OC cells stained with an isotype control, CD73-PE, or EpCAM-PE. Expression levels was analysed by flowcytometry. (B) Mean fluorescence intensity (MFI) of CD73 or EpCAM expression on patient-derived OC cells.

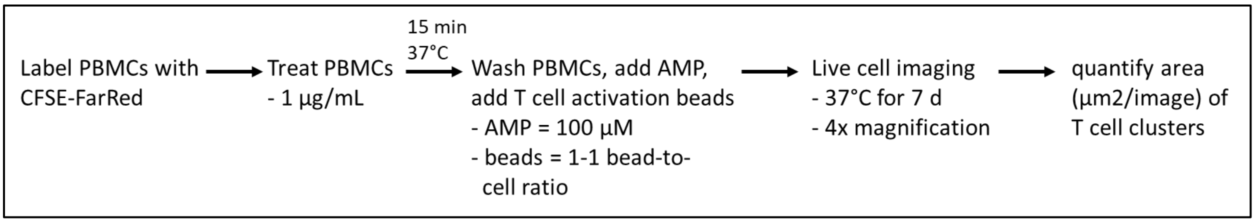

Supplementary Figure S7: Experimental procedure T cell proliferation
Peripheral Blood Mononuclear Cells (PBMCs) obtained from healthy donors were labeled with cell permeable fluorescent dye CFSE-FarRed according to manufacturer protocol. Next, CFSE-labeled PBMCs were treated (or not) with bsAb CD73xEpCAM (1 µg/mL) (or appropriate controls) for 15 min, washed, and activated by addition of T cell activation/expansion beads in a bead-to-cell ratio of 1-1 in medium supplemented (or not) with AMP (100 µM). Live cell imaging technology (IncuCyte) was used to evaluate the size of T cell clusters by taking pictures at 4 x magnification every 6 h for 7 d. The area (µm2/image) of activated T cell clusters was quantified using IncuCyte software 2019B.

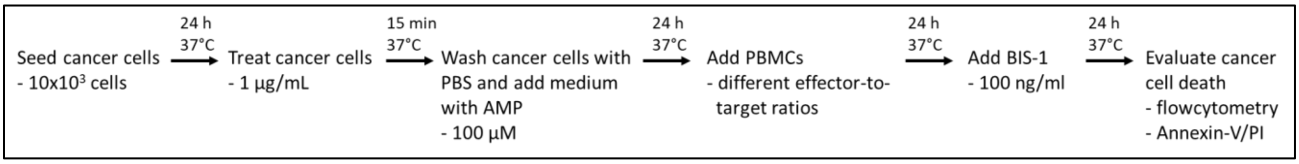

Supplementary Figure S8: Experimental procedure cancer cell elimination by T cells *Cancer cells were treated (or not) with bsAb CD73xEpCAM (1 µg/mL) (or appropriate controls) for 15 min, washed and incubated in medium supplemented (or not) with AMP (100µM) for at 37°C for 24 h. Next, freshly isolated PBMCs were added at different effector to target cell ratios to cancer cells, and then co-cultured at 37°C for 24 h. Subsequently, T cells present in the PBMC population were stimulated and re-directed to kill OvCAR3 cancer cells using EpCAM-directed/CD3-agonistic bispecific antibody BIS-1 at 37°C for 24 h. Apoptotic cancer cell death was assessed by flow cytometry.*

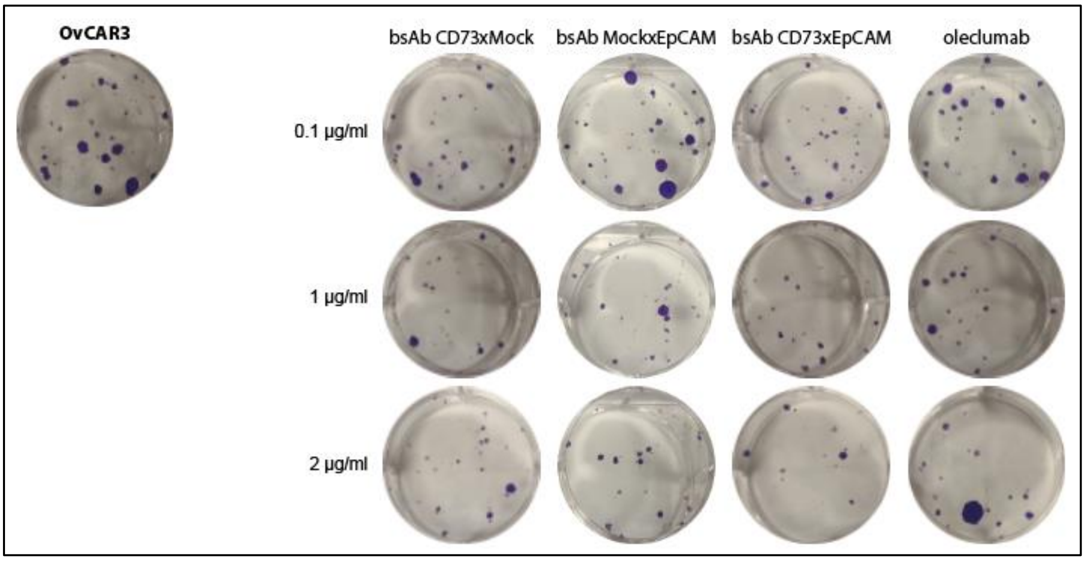

Supplementary Figure S9: bsAb CD73xEpCAM inhibits the colony-forming activity of OvCAR3 cancer cells
Representative pictures of OvCAR3 cell colonies after pretreatment (15 min) with bsAb CD73xEpCAM (0.1 – 2 µg/ml) (or controls) and subsequent culturing at 37 ºC for 14 d. Cell colonies were stained with crystal violet.
